# Clinical Application of Adaptive Immune Therapy in MSS Colorectal Cancer Patients

**DOI:** 10.3389/fimmu.2021.762341

**Published:** 2021-10-13

**Authors:** Danyang Wang, Hangyu Zhang, Tao Xiang, Gang Wang

**Affiliations:** ^1^ Department of Colorectal Surgery, First Affiliated Hospital, School of Medicine, Zhejiang University, Hangzhou, China; ^2^ Department of Medical Oncology, First Affiliated Hospital, School of Medicine, Zhejiang University, Hangzhou, China; ^3^ Department of Anorectal Surgery, Haiyan People’s Hospital, Jiaxing, China

**Keywords:** adaptive immune response, colorectal cancer, immune therapy, vaccines, CAR-T

## Abstract

Colorectal cancer (CRC) is one of the most common cancers worldwide. However, the treatment outcomes of immunotherapy in microsatellite-stable (MSS) CRC remain unsatisfactory. As the majority of CRC cases display a molecular MSS/mismatch repair-proficient (pMMR) profile, it is particularly meaningful to explore the clinical applications of adaptive immune therapy in MSS CRC patients. In this review, we summarized the therapeutic approaches of adoptive immune therapies, including cytokines, therapeutic cancer vaccines, adoptive T-cell therapy, and immune checkpoint inhibitors, in the treatment of MSS CRCs.

## Introduction

**Figure 1:**
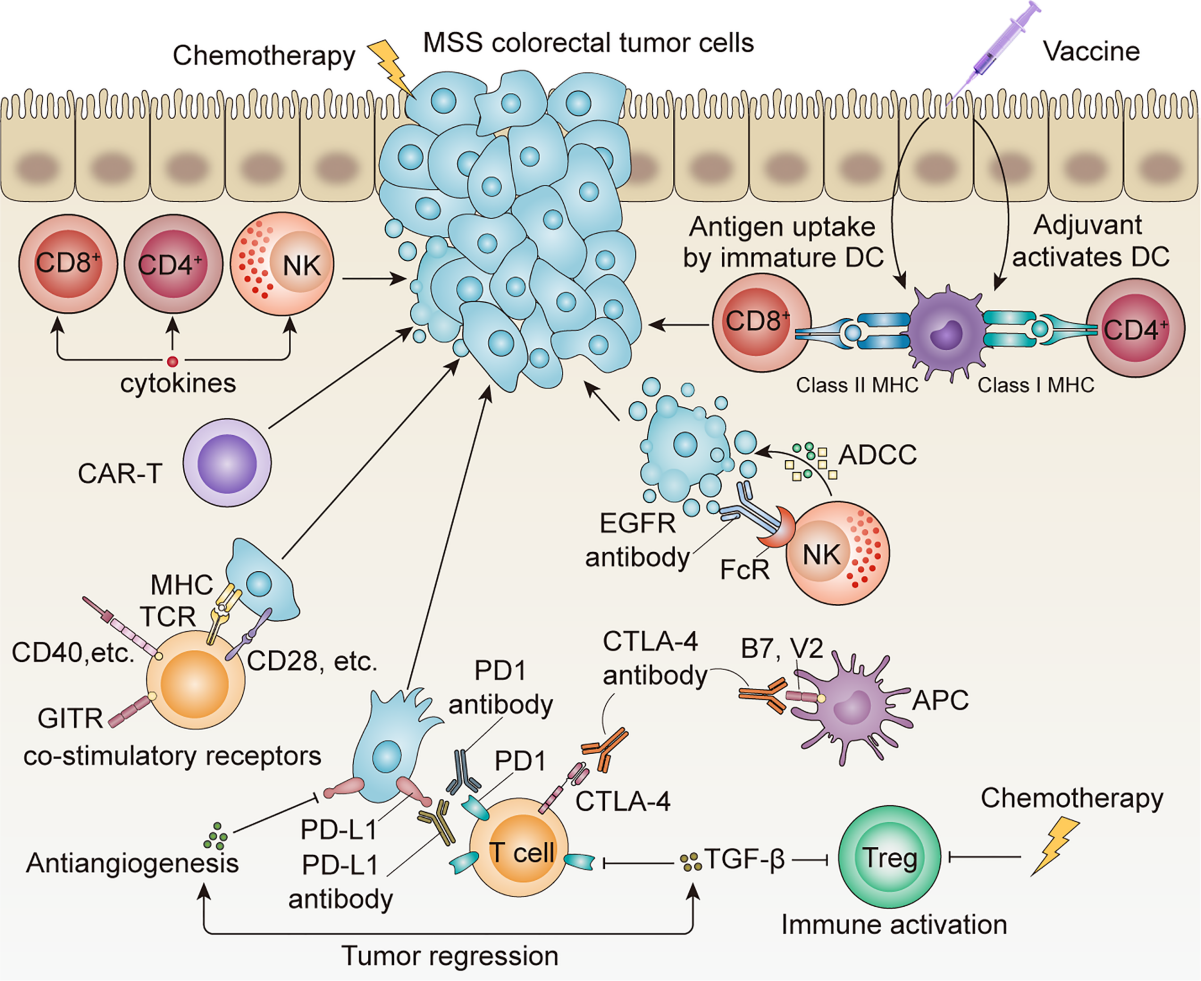
Application of multiple adaptive immunotherapies in MSS CRC. ICI monotherapy is often ineffective, and the combination of chemotherapy, antiangiogenesis therapy, anti-EGFR antibody, and other systemic therapies is expected to achieve better therapeutic outcomes.

Colorectal cancer (CRC) has a high incidence and is the third leading cause of cancer-related death worldwide (1). In recent years, despite the decrease in the overall incidence of CRC patients (2), the incidence of CRC in young patients has nearly doubled (3). Unfortunately, therapeutic options for colorectal cancer patients are still limited despite current systemic chemotherapy, targeted therapy, and local therapy tools. Recently, immunotherapy has become increasingly popular in the field of cancer therapy. The adaptive immune response plays an important role in the response to immunotherapy. The adaptive immune response always presents as specific and long-term memory, which result in durable responses. It has been confirmed that the presence of tumor-infiltrating lymphocytes (TILs) is associated with improved overall survival (4, 5), and the success of TILs in one patient with KRAS G12D mutation indicates the promise of adaptive immunotherapy in CRC patients (6). In addition, immune checkpoint inhibitors (ICIs) have shown great success in cancers such as melanoma and lung cancer (7–9). However, microsatellite-stable (MSS) metastatic colorectal cancer (mCRC) is usually considered to be a typical “cold” cancer that presents a poor response to immunotherapy. Indeed, MSS mCRC accounts for approximately 95% of all mCRC cases (10). It is important to explore how these patients can benefit from adaptive immunotherapy. This review highlights the current role of adaptive immune therapies, including cytokines, vaccines, cell therapies, and ICIs and their potential clinical applications in patients with MSS mCRC (**Figure 1**).

## Cytokines and Costimulatory Receptors

Extracellular cytokine components dynamically regulate the tumor microenvironment. The aberrant expression of cytokines is strongly correlated with the pathogenesis and progression of CRC (11). Cytokines have direct antitumor effects and have been used as cancer immunotherapies for decades, especially in metastatic melanoma and metastatic renal cell carcinoma (12, 13). Regarding CRC, evidence has shown that IL-6 can activate autophagy through the IL-6/JAK2/BECN1 pathway and promote chemotherapy resistance in CRC (14). One recent study showed that combined treatment with IL-2 and *Akkermansia muciniphila* leads to stronger antitumor efficacy in CRC patient-derived tumor tissue and proposed this novel therapeutic strategy with prospecting application in the future (15). Another study showed that IL-2 combined with TNFSF14 results in an increase in CD8+ central memory cells and a decrease in tumor size in preclinical exploration (16). Several clinical studies have proven that cytokines are tolerable safe, and more clinical trials are underway to explore their efficiency (11).

Another immunotherapy type is costimulatory receptors; among them, OX40 and 4-1BB are members of the TNF receptor and have the ability to activate stimulatory receptors and have shown promising antitumor effects in preclinical studies (17, 18). However, OX40 antibodies only show strong tumor suppressive effects when the tumor load is low, and further studies indicate that costimulatory receptors can improve treatment efficiency when combined with ICIs (19). A clinical trial (NCT 02179918) of multiple solid tumors was conducted on the combined treatment of a 4-1BB agonist plus a PD-1 inhibitor.

## Therapeutic Cancer Vaccines

Therapeutic cancer vaccines are diverse and include cell-based vaccines, protein/peptide vaccines, and genetic vaccines. Cancer vaccines are divided into tumor-associated antigen (TAA) vaccines and tumor-specific antigen vaccines. Currently, no significant breakthrough has been found in CRC vaccines, and no such vaccines have been approved for clinical treatment. However, as research continues, vaccines can present more potent antigens and activate both CD4^+^ and CD8^+^ T cells.

The most mature TAA vaccines in CRC are carcinoembryonic antigen (CEA) vaccines, melanoma-associated antigen (MAGE) vaccines, and intestinal protein guanylyl cyclase 2C (GUCY2C) vaccines. CEA is regularly overexpressed in CRC, but it is not cancer specific and is also expressed by normal epithelial cells (20). One of the first studies of CEA vaccines showed preliminary clinical benefit: two of 12 patients experienced dramatic tumor regression (21). Subsequently, studies suggested that different CEA peptide or mRNA vaccines can generate or boost specific T-cell responses and may provide clinical benefit in CRC patients (22, 23). However, the treatment outcome of CEA vaccines is not satisfactory because the clinical response rate does not exceed 17% (24) and consequently autoimmunity occurs. MAGE vaccines have the ability to induce an orchestrated CD4^+^ and CD8^+^ immune response. However, only one case report showed that MAGE-A4-H/K-HELP could induce Th1-dependent cells and decrease tumor growth and CEA tumor markers in colon cancer patients (25). Due to the poor response to a single vaccine, more combination treatment regimens of vaccines and personalized peptide vaccines have been studied and investigated in clinical trials. Most vaccines in these studies were combined with chemotherapies, such as 5-fluorouracil. Sato et al. (26) found that this combination therapy could actually maintain or augment immunological responses, but there was no confirmed decrease in tumor burden. GUCY2C vaccines focus on minimum residual disease in colon cancer patients and aim to reduce the recurrence rate (27). A preliminary phase I study suggested that the GUCY2C vaccine was safe and effective at inducing the CD8^+^ T-cell response in early-stage patients (28).

Regarding tumor-specific antigen vaccines, the number and quality of neoantigens are high (29). Tumor-specific vaccines have the advantage of recognizing cancer cells from normal cells and minimizing the risk of vaccination-reduced severe adverse events. However, MSS CRC usually shows fewer gene alterations than CRC with microsatellite instability-high (MSI-H) status or POLE mutations. In addition, heterogeneity exists and remains a challenge for developing a tumor-specific antigen vaccine. Neoantigens caused by KRAS are common in CRC, and peptides derived from mutated KRAS show certain anticancer activity in vaccination trials. In one case report, CRC lung metastasis patients quickly exhibited tumor regression after treatment with activated T cells recognizing G12D KRAS (6). Another clinical trial found that two out of seven patients were responsive after vaccine infusion (30).

Vaccines are able to activate cytotoxic T lymphocytes (CTLs), and the correlation between infiltrating lymphocytes and overall survival is significant in patients with MSS disease (31, 32). Therefore, a combination of ICIs and vaccines could be a new treatment strategy in the future. Preliminary animal studies have shown encouraging results (33), and several clinical trials are ongoing (**Table 1**).

**Table 1:** Current clinical studies of vaccines combined with ICIs.

Vaccination strategy	Therapy	No. of pts	Trial identifier
Nivolumab, MVA-BN-CV301	FOLFOX	78	NCT0357999
Atezolizumab, RO7198457		567	NCT03289962
Avelumab + Ad-CEA	FOLFOX, bevacizumab	81	NCT03050814

## Adoptive T-Cell Therapy

The patient’s T cells can target a specific antigen that is expressed on the surface of tumor cells. Thus, several types of T cells have been used for adoptive T-cell therapy, including TILs, chimeric antigen receptor (CAR)-engineered T cells and cancer-specific T-cell receptor-engineered T cells. These modified cells are reintroduced into the human body to fight against cancer cells and have achieved great success in hematological malignancies (34). However, the role of adoptive T-cell therapy in CRC is still unclear, and the treatment efficacy and safety remain to be verified in clinical studies.

CAR T-cell therapy has become increasingly popular in the last decade, and we are currently in the fourth generation of CAR T-cell immunotherapy (35). In the course of treatment, the patient’s immune cells are first harvested and cultured *in vitro*, and then CARs are artificially integrated into the T cells. Finally, the CAR T cells are infused back into the patient. The complexity of the entire process leads to the high cost of CAR T-cell therapy and high requirements for companies. Another significant problem with CAR T-cell therapy is toxicity. Cytokine release syndrome (CRS), which is not observed with traditional chemotherapy, is the main side effect and is caused by the release of inflammatory cytokines by continuously proliferating CAR T cells.

Multiple ongoing clinical trials of various potential targets of CAR T-cell therapy for colorectal cancer, including solid tumor targets such as GUCY2C, CEA, and TAG-72 and circulating tumor cell targets such as EpCAM, are shown in **Table 1**.

Different clinical studies have also employed different administration methods for CAR T cells, such as intravenous infusion, intraperitoneal infusion, and direct hepatic artery infusion (36). One of the first human CAR T-cell trials on metastatic colorectal cancer reported by Hege et al. (37) found that CART72 showed good safety and tolerance despite quick clearance from the blood. Another preclinical study (38) focusing on GUCY2C found certain effectiveness against mCRC in mouse models and in xenograft models of human CRC, and further human clinical trials are currently being recruited in China. Preliminary antitumor activity is also reflected in CAR T-133 cells, EpCAM CAR T cells, NKG2D CAR T cells, and HER2 CAR T cells (36, 39–42). Ongoing clinical trials of CAR T-cell therapy in the treatment of mCRC are shown in **Table 2**.

**Table 2:** Ongoing clinical trials of CAR T-cell therapy in CRC.

Target	Pathology	Study phase	No. of pts	Trial identifier
αPD-1-MSLN	mCRC	I	10	NCT04503980
CEA	Stage III and LM CRC	I	18	NCT04513431
CEA	mCRC	I	75	NCT02349724
CEA	mCRC	I	18	NCT03682744
CEA	mCRC	I/II	40	NCT04348643
EGFR	EGFR positive CRC	I/II	20	NCT03152435
MUC1	mCRC	II	20	NCT02617134
NKG2D	mCRC	I	10	NCT04550663

To date, there have been no particularly encouraging results about CAR T-cell therapy in mCRC. Although several promising CAR T-cell therapies have shown success in preclinical models, additional human data are still needed.

## Immune Checkpoint Inhibitors

The most well-studied immune checkpoints include PD-1, PD-L1, CTLA-4, LAG-3, and TIM-3. Despite the great success of the KEYNOTE 177 study (43) on MSI-H CRC, the majority of clinical trials of ICIs in MSS CRC have ended in failure. ICI monotherapy did not obtain positive results in MSS CRC patients in the KEYNOTE 016 and phase 2 CheckMate 142 trials. ICIs also failed as maintenance therapy in the MODUL study (44). PD-1 and CTLA-4 antibody combination therapy is a third-line treatment compared with best supportive care (45). The lack of mutant neoantigens, immune evasion, and neoangiogenesis are responsible for poor responses (46–49). In addition to treatment regimen exploration, the effect of metastatic sites on the efficacy of immunotherapy is also worthy of further study. Several studies have found that patients with liver metastasis often have a poor response to immunotherapy (50, 51). To overcome these problems, several new combination regimens have been explored. The rationale for combination therapy is that chemotherapy or targeted therapy can alter the tumor microenvironment (TME). Although different factors, such as low neoantigen burden and alterations in JAK/STAT pathways, may influence the treatment efficiency of ICIs, it is believed that the insensitivity of MSS CRC to immunotherapy could be reversed by altering the TME. The TME is a dynamic environment and is composed of cancer cells, extracellular matrix, immune cells, nutrients, and other components (52, 53).

Regarding combined checkpoint inhibitors, a phase I study found manageable safety and promising antitumor activity with the antilymphocyte activation gene (LAG-3) antibody plus pembrolizumab in MSS mCRC (54). Anti-TIM-3 antibody combined with anti-PD-1 antibody showed preliminary signs of antitumor activity in solid tumors (55).

### Combination With Chemotherapy

Chemotherapeutic drugs seem to reduce the levels of Tregs and increase the proliferation of homeostatic T cells (56). Oxaliplatin was proven to feasibly and substantially expand the response to ICIs in lung adenocarcinoma mouse models (57). Therefore, the phase II study POCHI (NCT 04262687) of the combination of oxaliplatin, capecitabine, bevacizumab, and pembrolizumab in CRC patients is ongoing.

Another study found that 5-fluorouracil could increase tumor visibility to immune cells, and the treatment response improved with sequential administration of 5-fluorouracil and ICIs compared with concurrent administration of ICIs with 5-fluorouracil (58).

### Combination With Radiotherapy

Radiotherapy can release neoantigens and inflammatory cytokines during treatment (59) and influence nonirradiated sites by activating the immune response (60), which is called the abscopal effect. However, the abscopal effect was observed in melanoma when ipilimumab was combined with localized radiotherapy (61). Regarding CRC, a phase II study enrolled 22 patients with MSS/mismatch repair-proficient (pMMR) CRC, and only one patient experienced regression at nonirradiated sites (62). There are a growing number of clinical trials exploring the combined efficiency of radiotherapy and immunotherapy. A phase II clinical trial of short-course radiotherapy combined with mFOLFOX6 chemotherapy and avelumab immunotherapy in Lebanon and Jordan enrolled 13 patients with MSS rectal cancer. One patient had preoperative progression, three patients achieved pCR, and three achieved near pCR (TRG1 score). That is, half of the patients achieved very significant tumor regression.

### Combination With Anti-EGFR Antibody

The combination of immunotherapy and EGFR antibodies can also enhance the immune response due to antibody-dependent cell-mediated cytotoxicity (ADCC) (63). One phase Ib/II trial of cetuximab combined with ICIs in nine mCRC patients showed that 67% of patients achieved stable disease (64). Another phase II trial of panitumumab plus ipilimumab and nivolumab in 56 refractory mCRC patients achieved a 35% 12-week response rate and 5.7-month progression-free survival (PFS) time (65).

### Combination With Antiangiogenesis Therapy

Immunotherapy is most commonly combined with VEGF inhibitors. Patients treated with antiangiogenic therapy had higher infiltration of CD4^+^FOXP3^+^ regulatory T cells and enhanced expression of checkpoint ligand programmed death-ligand 1 (PD-L1) in renal cell carcinoma specimens than the corresponding control (66). This phenomenon may be related to improved nutrient supply and oxygen demand with antiangiogenic therapy. However, an increasing number of studies have found that antiangiogenic drugs can modulate the TME, independent of their antiangiogenic effects. Regorafenib was found to have the ability to inhibit JAK1/2-STAT1 and MAPK signaling by targeting the RET-Src axis and to induce PD-L1 and IDO1 expression in melanoma (67). Another study found that regorafenib could reverse M2 macrophage polarization and the regorafenib dosage may be halved when combined with ICIs (68).

The REGNIVO study (69) showed a 36% overall response in 25 MSS CRC patients treated with regorafenib and nivolumab. However, among 48 patients in the REGOMUM study, no patient showed a partial or complete response. Therefore, this combination regimen still needs validation in a phase III study, and many clinical trials are ongoing.

### Combination With TGF-β Antibody

TGF-β upregulation plays an important role in the immune evasion of MSS mCRC. It has been reported that TGF-β can promote T-cell exclusion and block the acquisition of the Th1 effector phenotype (70). TGF-β inhibitors can render tumors susceptible to ICIs. Preclinical studies support the potential use of anti-PD-L1/TGF-β trap fusion proteins in clinical use, and related clinical trials are ongoing. Src homology 2 domain-containing tyrosine phosphatase 2 (Shp2) (71) and prostaglandin E2 (PGE2) receptor 4 (EP4) (72) are both potential strategies for enhancing the efficacy of immunotherapy for MSS mCRC by regulating immunosuppressive myeloid cells.

## Conclusion

Immunotherapy has a confirmed survival benefit for MSI-H mCRC. For MSS CRC, trials of monotherapy with ICIs and ICIs combined with other treatments, such as MEK inhibitors, have failed in the past decade. However, explorations of adaptive immune therapy in MSS CRC are ongoing. Clinical trials of cytokines, costimulatory receptors, CAR T cells and different combinations of anti-VEGF or anti-EGFR agents plus ICIs are ongoing. Preliminary data have indicated the great promise of combined adaptive immune therapy in MSS mCRC, and this type of treatment is expected to transform cold tumors into hot tumors.

## Author Contributions

All authors contributed to the study conception and design. Material preparation, data collection, and analysis were performed by DW, TX, and GW. The first draft of the manuscript was written by HZ, and all authors commented on previous versions of the manuscript. All authors read and approved the final manuscript.

## Conflict of Interest

The authors declare that the research was conducted in the absence of any commercial or financial relationships that could be construed as a potential conflict of interest.

## Publisher’s Note

All claims expressed in this article are solely those of the authors and do not necessarily represent those of their affiliated organizations, or those of the publisher, the editors and the reviewers. Any product that may be evaluated in this article, or claim that may be made by its manufacturer, is not guaranteed or endorsed by the publisher.
